# Identification of serum proteome signatures of locally advanced and metastatic gastric cancer: a pilot study

**DOI:** 10.1186/s12967-015-0668-9

**Published:** 2015-09-17

**Authors:** Agata Abramowicz, Anna Wojakowska, Agnieszka Gdowicz-Klosok, Joanna Polanska, Pawel Rodziewicz, Pawel Polanowski, Agnieszka Namysl-Kaletka, Monika Pietrowska, Jerzy Wydmanski, Piotr Widlak

**Affiliations:** Maria Sklodowska-Curie Memorial Cancer Center and Institute of Oncology, Gliwice, Poland; Silesian University of Technology, Gliwice, Poland; Institute of Bioorganic Chemistry, Polish Academy of Science, Poznan, Poland

**Keywords:** Stomach cancer, Early diagnosis, Neoplasms, Proteomics, Biological markers

## Abstract

**Background:**

The gastric cancer is one of the most common and mortal cancer worldwide. The initial asymptomatic development and further nonspecific symptoms result in diagnosis at the advanced stage with poor prognosis. Yet, no clinically useful biomarkers are available for this malignancy, and invasive gastrointestinal endoscopy remains the only reliable option at the moment. Hence, there is a need for discovery of clinically useful noninvasive diagnostic and/or prognostic tool as an alternative (or complement) for current diagnostic tools. Here we aimed to search for serum proteins characteristic for local and invasive gastric cancer.

**Methods:**

Pre-treatment blood samples were collected from patients with diagnosed gastric adenocarcinoma at the different stage of disease: 35 patients with locally advanced cancer and 18 patients with metastatic cancer; 50 healthy donors were also included as a control group. The low-molecular-weight fraction of serum proteome (i.e., endogenous peptidome) was profiled by the MALDI-ToF mass spectrometry, and the whole proteome components were identified and quantified by the LC–MS/MS shotgun approach.

**Results:**

Multicomponent peptidome signatures were revealed that allowed good discrimination between healthy controls and cancer patients, as well as between patients with locally advanced and metastatic cancer. Moreover, a LC–MS/MS approach revealed 49 serum proteins with different abundances between healthy donors and cancer patients (predominantly proteins associated with inflammation and acute phase response). Furthermore, 19 serum proteins with different abundances between patients with locally advanced and metastatic cancer were identified (including proteins associated with cytokine/chemokine response and metabolism of nucleic acids). However, neither peptidome profiling nor shotgun proteomics approach allowed detecting serum components discriminating between two subgroups of patients with local disease who either developed or did not develop metastases during follow-up.

**Conclusions:**

The molecular differences between locally advanced and metastatic gastric cancer, as well as more obvious differences between healthy individuals and cancer patients, have marked reflection at the level of serum proteome. However, we have no evidence that features of pre-treatment serum proteome could predict a risk of cancer dissemination in patients treated due to local disease. Nevertheless, presented data confirmed potential applicability of a serum proteome signature-based biomarker in diagnostics of gastric cancer.

**Electronic supplementary material:**

The online version of this article (doi:10.1186/s12967-015-0668-9) contains supplementary material, which is available to authorized users.

## Background

Gastric cancer is the fourth most common cancer and the second leading cause of cancer-related death worldwide. This cancer especially afflicts populations of East Asia, Eastern Europe, and parts of Central and South America, and the morbidity rate is twice higher for men than for women [1]. The malignancy is associated with nonspecific symptoms or even asymptomatic development in its early stages, which often results in diagnosis at advanced stages. Stage of gastric cancer strongly correlates with poor prognosis. According to the National Cancer Data Base report, 5-year survival rate for stage IA was 78 % and it dropped substantially at each stage to about 7 % for patients diagnosed with stage IIIB or stage IV disease [2]. Thus, early diagnosis of gastric cancer might radically increase efficacy of treatment and improve prognosis for this fatal illness. At present the most efficient diagnostic tool for detection of gastric cancer remains a gastrointestinal endoscopy, yet this invasive technique is not suitable for large-scale screening. Unfortunately, there is no alternative non-invasive biomarkers available, because the commonly used gastrointestinal tumor markers like CEA, CA 19-9 or CA 72-4 are insufficient for early diagnosis of this cancer due to their low sensitivity and specificity (20–30 %) [3–5]. Majority of gastric cancer cases (above 90 %) are classified as adenocarcinomas. More recently four molecular subtypes of gastric adenocarcinoma were distinguished based on genomic profiling delivered thanks to the Cancer Genome Atlas project [6]. However, the knowledge on molecular heterogeneity and biology of this cancer, including its development and mechanisms of progression, remains rather limited yet. Hence, an urgent need for identification of clinically relevant biomarkers relates not only the early diagnosis but also prognosis and prediction of treatment outcome.

Clinical proteomics is an important approach to discovery of biomarkers of gastric cancer [7]. It is generally accepted that blood proteome is a promising source of novel biomarkers of this cancer, including particularly valuable markers for early detection of the disease and monitoring of response to the treatment [8]. Mass spectrometry-based profiling of the low-molecular-weight fraction of serum proteome, so called endogenous peptidome, revealed multi-peptide signatures with potential applicability in classification and diagnosis of different cancer types [9–13]. A few works have been published that explored MALDI/SELDI-based profiling of serum/plasma petidome for diagnosis of gastric cancer, which proposed peptide signatures that allowed discriminating healthy donors and patients with gastric cancer, or signatures associated with a course of a disease [14–22]. Several components of such signatures were further identified as fragments of KNG1 [18], APOC1 and APOA2 [19], SAA [20], TBB5 and TYB4 [22] or FIBA [23, 24]. More recently, a panel of biomarkers composed of serum proteins pre-selected based on preclinical mouse model (afamin, clusterin, VDBP and haptoglobin) has been validated to discriminate between gastric cancer patients and patient with benign gastric diseases [25]. Nevertheless, none of proposed serum proteome signatures of gastric cancer has been widely accepted and applied in clinical practice yet.

Here we aimed to characterize proteome features of pre-treatment serum associated with risk of metastasis of gastric cancer. Two types of proteomic analyses were performed: (1) the low-molecular-weight fraction of serum proteome was profiled by the MALDI-ToF mass spectrometry, (2) the whole proteome components were identified and quantified by LC–MS/MS after digestion with trypsin (a “shotgun proteomics” approach). Groups of previously untreated patients with locally advanced gastric cancer and metastatic disease were enrolled to this study (a matched group of healthy individuals was analyzed as a reference); such comprehensive proteomic analysis was performed in a group of Caucasians patients with gastric cancer for the first time. Serum proteome signature that differentiated between patients with locally advanced cancer and metastatic cancer was detected in this pilot study, yet features specific for patients with locally advanced disease at time of diagnosis who eventually developed metastases were not observed at this level.

## Methods

### Characteristics of patient groups

Fifty-three patients with previously untreated biopsy-proven gastric adenocarcinoma were qualified into this study: 35 patients with locally advanced cancer, including 16 patients with metastasis developed during therapy or follow-up and 19 patients with no detected metastasis during follow-up, and 18 patients with metastatic cancer. The latter group consisted of four patients with distant spread to single organ and 14 patients with spread to multiple organs; involved organs included peritoneum (13 cases), liver (eight cases), lung (three cases), other locations (eight cases). In general, inclusion criteria involved: Eastern Cooperative Oncology Group (ECOG) performance status of 0–2, age 20–85 years, serum creatinine level <1.5 mg/dl, serum bilirubin level <2.0 mg/dl, a granulocyte count >1500 cells/μl and a platelet count >100,000 cells/μl, while exclusion criteria involved: previous malignancy, previous surgery, radiotherapy or chemotherapy. The pretreatment staging based on physical examination, esophagogastroscopy with biopsies, CT of the abdomen and chest examination by X-ray or CT. Fifty sex- and age-matched disease-free donors were included as a control group. All study participants were Caucasians (~65 % men) with the age at the range 34–74 years. Table 1 shows more detailed information about analyzed groups. The study was approved by the appropriate Ethics Committee, and all persons who were taking part in this study provided informed consent indicating their conscious and voluntary participation.

**Table 1:** Characteristics of donor groups enrolled into the study

Group/parameter	Healthy control	Cancer patients (all cases)	Patients with locally advanced cancer		Patients with metastatic cancer
			No spread	Spread	
Number (n)	50	53	19	16	18
Sex (M/F)	30/20	37/16	12/7	13/3	12/6
Age (years)	28–60 (median 50)	34–74 (median 59)	36–70 (median 59)	34–73 (median 58)	35–74 (median 60)
Tumor location					
Upper third	–	15 (28 %)	3 (16 %)	6 (37 %)	6 (33 %)
Middle third	–	31 (59 %)	13 (68 %)	9 (57 %)	9 (50 %)
Lower third	–	7 (13 %)	3 (16 %)	1 (6 %)	3 (17 %)
Histological grade					
G1–G2	–	16 (30 %)	10 (53 %)	3 (19 %)	3 (17 %)
G3	–	29 (55 %)	8 (42 %)	11 (69 %)	10 (56 %)
Not specified	–	8 (15 %)	1 (5 %)	2 (12 %)	5 (27 %)
Primary tumor					
cT1–T3	–	50 (94 %)	19 (100 %)	16 (100 %)	15 (83 %)
cT4	–	3 (6 %)	0 (0 %)	0 (0 %)	3 (17 %)
Lymph node					
cN0	–	20 (38 %)	13 (68 %)	5 (31 %)	2 (11 %)
cN1–N3	–	33 (62 %)	6 (32 %)	11 (69 %)	16 (89 %)
Metastasis (initial)					
cM0	–	35 (66 %)	19 (100 %)	16 (100 %)	0 (0 %)
cM1	–	18 (34 %)	0 (0 %)	0 (0 %)	18 (100 %)

Group of patients with locally advanced cancer at time of diagnosis were further split into subgroup where either no spread (control) or consecutive cancer dissemination/spread (distant metastasis) was detected

### Preparation of serum samples

Pre-treatment blood was collected into a 5 ml Vacutainer Tube (Becton–Dickinson), incubated for 30 min at room temperature to allow clotting and then centrifuged at 1000*g* for 10 min to remove the clot. The serum was aliquoted and stored at −70 °C until use.

### Profiling of the low-molecular-weight fraction of serum proteome

Before analysis samples were diluted 1:5 with buffer containing 20 % acetonitrile (ACN) and 25 mM ammonium bicarbonate, and then filtered by centrifugation through Amicon Ultra units (50 kDa cut-off) for removing the abundant high-molecular weight proteins, particularly albumin. Immediately before analysis samples were desalted and concentrated by loading onto ZipTip C18 microcolumns (EMD Millipore), and then eluted with 1 μl of matrix solution (saturated solution of alpha-cyano-4-hydroxy-cinnamic acid in 30 % ACN/H_2_O and 0.1 % TFA) directly onto the 800 μm AnchorChip™ (Bruker Daltonics) plate. The analysis was done by using an UltrafleXtreme MALDI-ToF mass spectrometer (Bruker Daltonics); the analyzer worked in the linear mode, and positive ions were recorded in the mass range between 1000 and 12,000 Da. The samples were spotted in duplicate and for each spot two spectra were acquired. Mass calibration was performed after every four samples using Protein Calibration Standard I (Bruker Daltonics). Randomization in blocks was used in spectra registration to avoid a possible batch effect. Afterwards the raw data was exported to TXT files and the spectral components were preprocessed using bioinformatics algorithms created in our group, which included alignment, detection and removal of outlier profiles by Dixon’s Q test (single spectra were removed from about 5 % of samples), averaging of technical repeats, baseline removal and normalization of the total ion current. The spectra smoothing, peak picking, binning and statistical analysis were performed using Spectrolyzer software (version 1.0.21.3590, MedicWave).

### LC–MS/MS analysis of serum proteome components

Serum samples were reduced with 5 mM dithiothreitol for 5 min at 95 °C, then alkylated with 10 mM iodoacetamide for 20 min in darkness at room temperature, and afterwards digested overnight at 37 °C with trypsin (Promega). The analysis was performed on Dionex UltiMate 3000 RSLC nanoLC System connected to Q Exactive Orbitrap mass spectrometer (Thermo Fisher Scientific); each sample was analyzed separately. Tryptic peptides (2.5 µg of peptides) were separated on reverse phase Acclaim PepMap RSLC nanoViper C18 column (75 µm × 25 cm, 2 µm granulation) using the acetonitrile gradient (from 4 to 60 %, in 0.1 % formic acid) at 30 °C and a flow rate of 250 nL/min (for 230 min). The spectrometer was operating in the data-dependent MS/MS mode with survey scans acquired at a resolution of 70,000 at m/z 200 Da in MS mode and 17,500 at m/z 200 Da in MS2 mode, respectively. The spectra were recorded in the scan m/z range 300–2000 in the positive ion mode. Higher energy collisional dissociation (HCD) ion fragmentation was performed with normalized collision energies set to 25. Protein identification was performed using Swiss-Prot human database with a precision tolerance 10 ppm for peptide masses and 0.05 Da for fragment ion masses. The abundances of identified proteins were estimated using MaxQuant 1.4.1.1 software.

### Statistical and bioinformatics analyses

For each component of MALDI mass profiles the comparison between groups of donors was performed using the Student’s t test after logarithmic transformation of data. Multi-component classifiers were built and tested with the SVM-based approach using Spectrolyzer software (version 1.0.21.3590, MedicWave). Significance of differences in abundances of proteins quantified by LC–MS/MS were assessed using the t test or the Mann–Whitney test depending on normality of data (type of distribution was estimated using the Shapiro–Wilk test, the Lilliefors test and the F test for homogeneity of variances), and the Nemenyi test for pairwise comparisons. In general, p = 0.05 was selected as a statistical significance threshold except for MALDI profiling where the Bonferroni correction for multiple testing was applied. The Empirical Proteomic Ontology Knowledge Base (EPO-KB), which annotates registered m/z values to known peptide/proteins [26], was employed to assign hypothetical identification of the spectra components (0.5 % mass accuracy limit was allowed). List of genes corresponding to identified proteins was annotated at GO terms using gProfiler (http://biit.cs.ut.ee/gprofiler/); the significance of the term over-representation was assessed using the hypergeometric distribution test. In order to visualize functional relationships between identified proteins corresponding genes were annotated at the GeneMANIA Cytoscape plugin for pathway interaction networks (http://pages.genemania.org/plugin/).

## Results

**Fig. 1:**
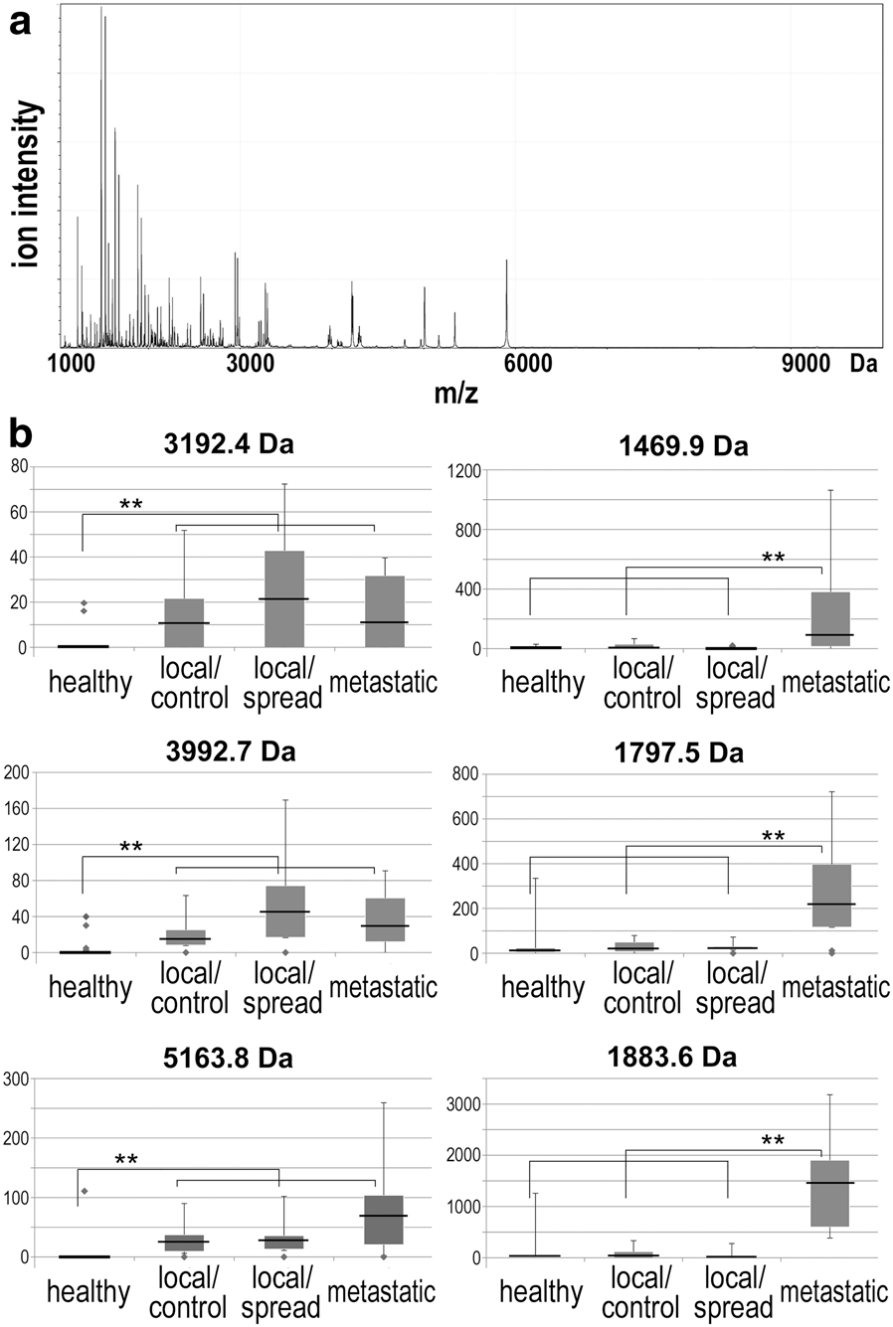
Profile of endogenous serum peptidome of patients with gastric cancer. **a** Average mass spectrum in the range of 1000–10,000 Da. **b** Examples of serum peptidome components, which abundances were different between samples from healthy controls and different groups of patients with stomach cancer. *Boxplots* show minimum, lower quartile, median, upper quartile, maximum values, and outliers; *asterisks* marked significant differences (p < 0.05 with the Bonferroni correction)

Mass profiles of the serum endogenous peptidome (the low-molecular-weight fraction of serum proteome) were characterized by MALDI-ToF spectrometry in the whole group of 53 patients with gastric cancer and 50 matched healthy donors. This analysis allowed us to asses overall degree of differences and similarities between subgroups of analyzed individuals. In general, 255 spectral components (peptide ions) were distinguished in the analyzed mass range (Fig. 1a), and abundances of 101 components revealed statistically significant variation among compared groups (after the Bonferroni correction against multiple testing). Table 2 presents numbers of serum peptidome components that differentiated particular groups of donors. The major differences in abundances of specific serum components were observed between cancer patients and healthy donors (about 39 % of registered components reveled statistically significant differences). Large differences were also observed between patients with locally advanced cancer (all cases) and patients with metastatic cancer (about 9 % of registered components reveled statistically significant differences); this is noteworthy that similar numbers of differentiating components were observed when patients with metastatic cancer were compared with both subgroups with local disease separately (i.e., group where distant spread of cancer was detected during follow-up and group without evidence of disease). Coherently, well performing classifiers could be built based on features of serum peptidome that separated compared groups of healthy donors and patients with locally advanced or metastatic cancer (the AUC measure for SVM-based classifier was above 90 % in each case). In marked contrast, no statistically significant difference was detected when two subgroups of patients with locally advanced cancer (who either developed or not developed metastasis during follow-up) were compared (the AUC of SVM-based classifier was below 50 %). Figure 1b presents examples of serum peptidome components with significantly different abundances among compared groups. This is noteworthy that among petidome components that differentiated both healthy controls from cancer patients and patients with locally advanced cancer from patients with metastatic disease there were several components that putatively corresponded to fragments of fibrinopeptide A (FIBA). These included components with registered m/z value 1088.7, 5903.5 and 5916.6 Da significantly downregulated in serum of cancer patients, and components 1469.9 and 1626.0 Da with markedly higher abundances in serum of patients with metastatic disease than in patients with locally advanced cancer (see Fig. 1b; Additional file 1: Table S1). We concluded that molecular differences between locally advanced and metastatic gastric cancer (as well as differences between healthy individuals and patients with gastric cancer in general) have reflection at the level of serum peptidome. However, features of peptidome of pre-treatment serum could be unlikely applied for prognosis of the disease spread during/after the treatment.

**Table 2:** Numbers of serum peptidome components with abundances different between compared groups of individuals

Groups/differences	Control vs. cancer (all cases)	Locally advanced vs. metastatic cancer	Local/no spread vs. metastatic cancer	Local/spread vs. metastatic cancer	Local/no spread vs. local/spread cancer
n	50 vs. 53	35 vs. 18	19 vs. 18	16 vs. 18	19 vs. 16
p < 0.05	182	88	74	75	11
FDR	7 %	14 %	17 %	17 %	100 %
p < 0.05/Bonferroni	101	23	11	16	0
AUC (SVM)	0.94	0.91	0.92	0.97	0.48

Shown are numbers of differentiating components that reached threshold of statistical significance p = 0.05 (with corresponding FDR estimation) or threshold strengthened with the Bonferroni correction, and power of SVM classifier built of peptidome components (characterized by the AUC value)

**Fig. 2:**
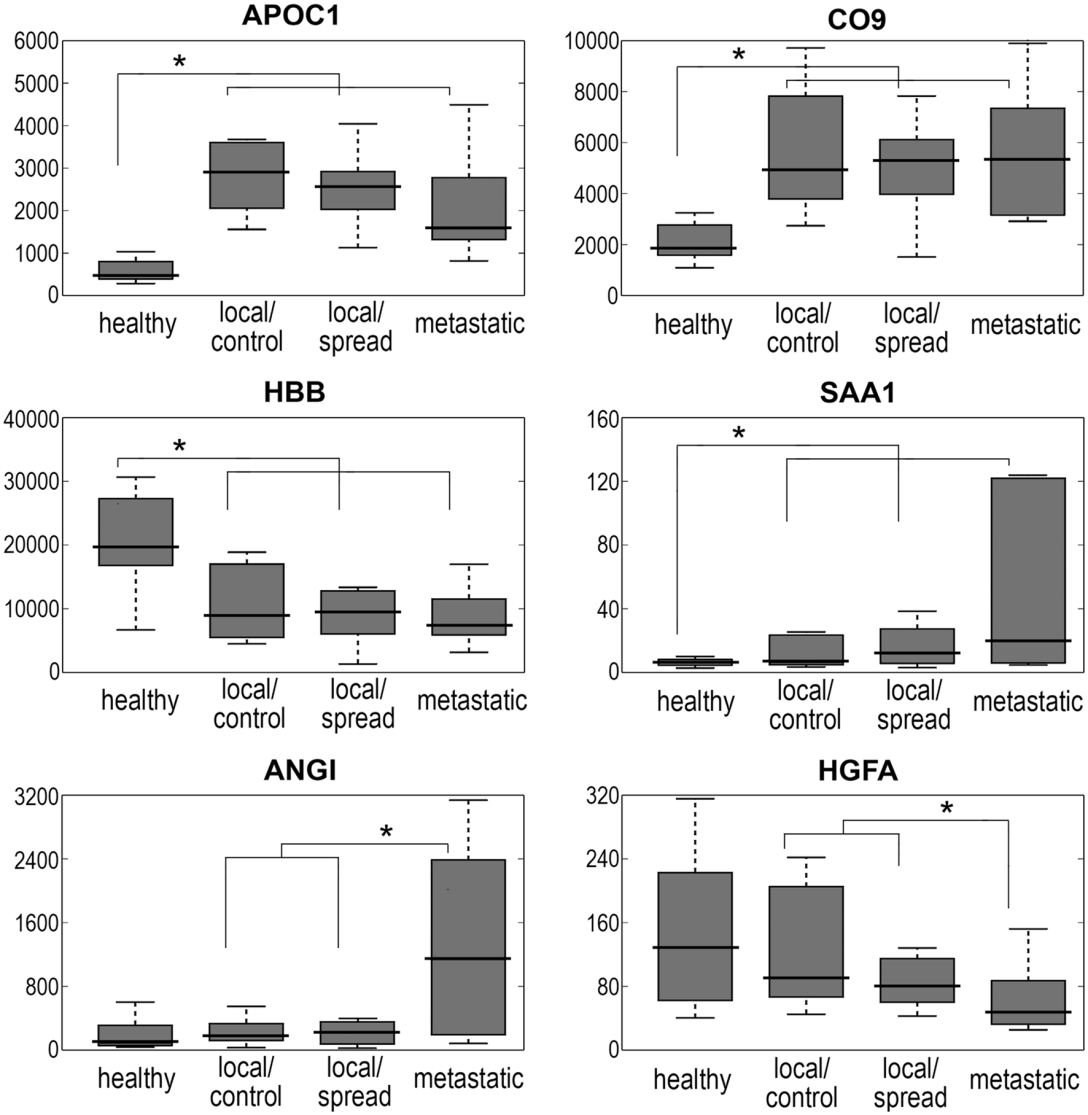
Examples of serum proteins that discriminated groups of donors. *Boxplots* show minimum, lower quartile, median, upper quartile and maximum values; *asterisks* marked significant differences (p < 0.05)

In the second step we selected samples of 12 healthy donors and ten patients from each cancer subgroup to secure similar age (medians about 56 years) and proportion of sexes (ca. 80 % of males) in compared subgroups. These samples were used for further analyses based on the shotgun LC–MS/MS approach. In general, about 450 proteins were identified in analyzed serum samples. Levels of 234 serum proteins were quantified in samples collected from each of 42 individuals, including 129 unique serum proteins not related to immunoglobulins (105 immunoglobulins and Ig-related proteins, as well as putative uncharacterized proteins, were excluded from further analyses); complete data are presented in the Additional file 1: Table S2. There were 49 serum proteins with abundances different between healthy donors and patients with gastric cancer (p value <0.05; estimated FDR value = 13 %): 41 proteins were upregulated while eight proteins were downregulated in blood of cancer patients (proteins listed in Table 3; examples in Fig. 2). This is noteworthy that similar patterns of cancer-related upregulation or downregulation of serum proteins was observed in all there subgroups of cancer patients (even though smaller size of analyzed groups might reduce statistical significance of differences; see Additional file 1: Table S3). Moreover, there were 19 serum proteins with abundances different between patients with locally advanced and metastatic cancer (p value <0.05; estimated FDR value = 34 %): three proteins were upregulated while 16 proteins were downregulated in blood of patients with metastatic disease. This is noteworthy that abundances of C-reactive protein and angiogenin generally upregulated in cancers samples further increased in metastatic samples, while abundances of carbonic anhydrase 1 generally downregulated in cancer samples further decreased in metastatic samples (Table 3). Furthermore, patterns of differences between samples from patients with metastatic cancer and all patients with local disease were retained when two smaller subgroups of patients with locally advanced cancer were pairwise compared with metastatic cancer (see Additional file 1: Table S4). On the other hand, abundance of only one protein (antithrombin-3) showed statistically significant difference when both subgroups of patients with locally advanced cancer were compared (abundance of this protein was the highest in group of patients with local disease who do not spread during follow-up). We concluded that multi-protein signature could be identified for classification of pre-treatment serum samples of gastric cancer patients, who suffered from either locally advanced or metastatic disease. However, features of serum proteome could not distinguish patients with local disease that spread during follow-up after the treatment.

**Table 3:** Differentiating serum proteins

Protein name	Protein full name	Gene name	Control/cancer		Local/metastatic	
			Ratio	p value	Ratio	p value
A1AG1	Alpha-1-acid glycoprotein 1	ORM1	*0.67*	*0.0008*	1.00	0.5824
A1AG2	Alpha-1-acid glycoprotein 2	ORM2	*0.68*	*0.0006*	1.00	0.7414
A1AT	Alpha-1-antitrypsin	SERPINA1	*0.50*	*<0.0001*	1.03	0.6441
A1BG	Alpha-1B-glycoprotein	A1BG	*0.76*	*0.0072*	1.04	0.5824
A2GL	Leucine-rich alpha-2-glycoprotein	LRG1	*0.46*	*0.0016*	0.80	0.4414
AACT	Alpha-1-antichymotrypsin	SERPINA3	*0.54*	*0.0007*	0.97	0.9124
ADIPO	Adiponectin	ADIPOQ	*0.41*	*0.0092*	1.60	0.3442
AFAM	Afamin	AFM	*1.30*	*0.0355*	1.06	0.5824
ANGI	Angiogenin	ANG	*0.30*	*0.0465*	*0.24*	*0.0294*
APOA1	Apolipoprotein A-I	APOA1	*0.47*	*<0.0001*	*1.31*	*0.0235*
APOC1	Apolipoprotein C-I	APOC1	*0.25*	*<0.0001*	1.52	0.1183
APOC3	Apolipoprotein C-III	APOC3	*1.82*	*0.0108*	1.20	0.5824
APOE	Apolipoprotein E	APOE	*0.62*	*0.0040*	1.01	0.6441
APOF	Apolipoprotein F	APOF	*0.64*	*0.0085*	0.80	0.5235
APOM	Apolipoprotein M	APOM	0.92	0.4275	*1.51*	*0.0263*
C1S	Complement C1s subcomponent	C1S	*0.70*	*0.0016*	1.22	0.0748
CAH1	Carbonic anhydrase 1	CA1	*2.96*	*0.0173*	*2.41*	*0.0143*
CBG	Corticosteroid-binding globulin	SERPINA6	*0.53*	*0.0117*	1.08	0.7749
CD14	Monocyte antigen CD14	CD14	*0.56*	*0.0033*	1.21	0.1658
CERU	Ceruloplasmin	CP	*0.67*	*0.0013*	1.05	0.6129
CFAB	Complement factor B	CFB	*0.61*	*0.0006*	1.11	0.6441
CO2	Complement C2	C2	*0.64*	*0.0475*	1.57	0.0679
CO4A	Complement C4-A	C4A	*0.87*	*0.0435*	0.94	0.7749
CO4B	Complement C4-B	C4B	*0.73*	*0.0127*	0.99	0.5526
CO5	Complement C5	C5	*0.77*	*0.0085*	0.91	0.8776
CO6	Complement component C6	C6	*0.64*	*0.0013*	0.97	0.8431
CO8G	Complement component C8 gamma	C8G	*0.66*	*0.0137*	1.12	0.4679
CO9	Complement component C9	C9	*0.42*	*<0.0001*	0.93	0.9124
CRP	C-reactive protein	CRP	*<0.01*	*0.0389*	*0.15*	*0.0414*
CXCL7	Platelet basic protein	PPBP	0.83	0.1516	*1.53*	*0.0030*
FETUA	Alpha-2-HS-glycoprotein	AHSG	0.90	0.3095	*1.34*	*0.0068*
FHR1	Complement factor H-related prot. 1	CFHR1	0.66	0.0725	*1.30*	*0.0366*
GPX3	Glutathione peroxidase 3	GPX3	0.92	0.6065	*1.47*	*0.0453*
HBA	Hemoglobin subunit alpha	HBA1	*2.02*	*0.0016*	1.18	0.5824
HBB	Hemoglobin subunit beta	HBB	*2.26*	*0.0005*	1.11	0.6441
HBD	Hemoglobin subunit delta	HBD	*4.59*	*0.0030*	1.02	0.9608
HEMO	Hemopexin	HPX	*0.77*	*0.0061*	1.00	0.9124
HGFA	Hepatocyte growth factor activator	HGFAC	1.57	0.0868	*1.74*	*0.0366*
HGFL	Hepatocyte growth factor-like prot.	MST1	1.40	0.4048	*3.28*	*0.0156*
HPT	Haptoglobin	HP	*0.68*	*0.0014*	1.02	0.7749
IC1	Plasma protease C1 inhibitor	SERPING1	*3.36*	*0.0001*	*0.21*	*0.0030*
ITIH1	Inter-alpha-trypsin inhib. heavy ch. 1	ITIH1	*0.77*	*0.0465*	1.06	0.2099
ITIH3	Inter-alpha-trypsin inhib. heavy ch. 3	ITIH3	*0.45*	*0.0001*	1.05	0.4414
LBP	Lipopolysaccharide-binding protein	LBP	*0.42*	*0.0004*	0.78	0.7084
LG3BP	Galectin-3-binding protein	LGALS3BP	*0.23*	*0.0006*	1.29	0.8776
PGRP2	*N*-acetylmuramoyl-l-alanine amidase	PGLYRP2	*0.82*	*0.0497*	1.15	0.4157
PLF4	Platelet factor 4	PF4	1.00	0.9667	*1.41*	*0.0366*
PON1	Serum paraoxonase/arylesterase 1	PON1	0.88	0.4606	*1.42*	*0.0442*
PROP	Properdin	CFP	*1.45*	*0.0028*	1.11	0.9825
PROZ	Vitamin K-dependent protein Z	PROZ	0.93	0.6864	*2.08*	*0.0186*
S10A9	Protein S100-A9	S100A9	*0.11*	*0.0054*	0.74	0.2526
SAA1	Serum amyloid A-1 protein	SAA1	*0.04*	*0.0407*	0.49	0.2099
SAA2	Serum amyloid A-2 protein	SAA2	*<0.01*	*0.0389*	0.44	0.3069
SEPP1	Selenoprotein P	SEPP1	1.72	0.1292	*1.52*	*0.0186*
SHBG	Sex hormone-binding globulin	SHBG	*0.20*	*0.0329*	0.67	0.1968
THBG	Thyroxine-binding globulin	SERPINA7	*0.70*	*0.0309*	0.80	0.8088
TSP1	Thrombospondin-1	THBS1	0.74	0.1363	*1.49*	*0.0436*
VTDB	Vitamin D-binding protein	GC	*0.81*	*0.0160*	*1.24*	*0.0235*
VTNC	Vitronectin	VTN	*0.82*	*0.0394*	1.15	0.1083

Showed are proteins, which abundances were different between samples from healthy controls and patients with stomach cancer (all cases), or between patients with locally advanced and metastatic cancer. Differences (ratios of the mean abundances) that passed the threshold of statistical significance (p < 0.05) are marked in italics characters

**Fig. 3:**
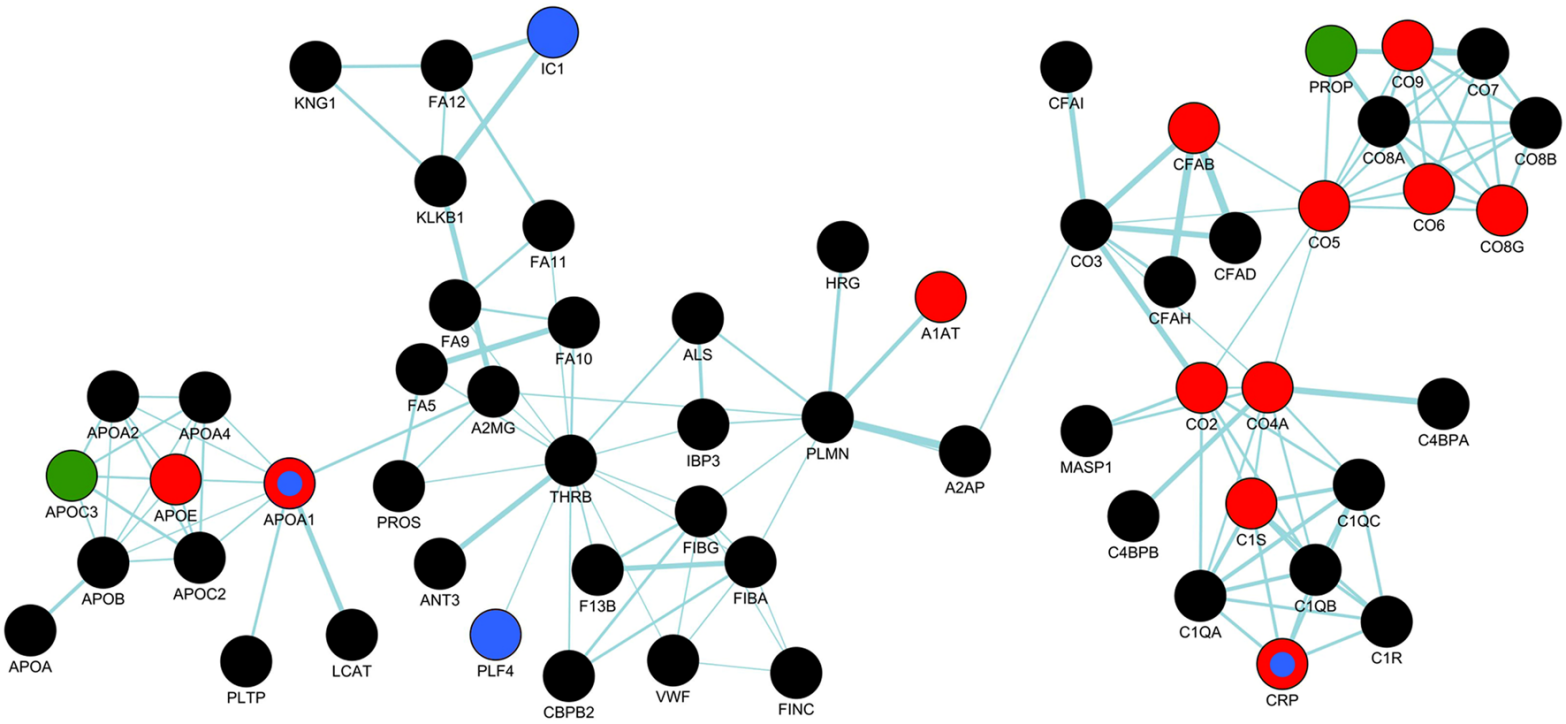
Network of functional interaction between serum proteins associated with stomach cancer. *Red* and *green* *circles* represent proteins upregulated and downregulated, respectively, in samples from cancer patients; *blue circles* represent proteins with significantly different abundances in samples of patients with locally advanced and metastatic disease (*black circles* represent other quantified proteins involved in interactions)

To identify molecular processes associated with serum proteins characteristic for gastric cancer, corresponding genes were annotated at the Gene Ontology database (Ig-related proteins were excluded from the analysis) (see Additional file 1: Table S5). There were 59 over-represented GO terms associated with proteins differentiating cancer patients from healthy donors. Among them dominated terms associated with different aspects of defense, inflammation and immune response (23 terms). Network of functional interactions was built based on the GeneMANIA Cytoscape tool to illustrate confirmed interactions between serum proteins characteristic for cancer patients. Figure 3 shows such network and its overlap with functional group of proteins associated with inflammatory/acute phase response and/or complement activation (CRP, C1S, CO2, CO4A, CO5, CO6, CO8G, CO9, CFAB, PROP). Moreover, there were 57 over-represented GO terms associated with proteins differentiating patients with locally advanced and metastatic disease. Among them dominated terms associated with metabolism of phosphorus and nucleic acids (16 terms) and with response to cytokines/chemokines and leukocyte migration (13 terms) (see Additional file 1: Table S6). We concluded that serum proteome signature that differentiated gastric cancer patients from healthy donors consisted of proteins primarily involved in cancer-type-nonspecific processes associated with inflammation and immune response (even though Ig-related proteins were excluded from analysis). On the other hand, serum proteome signature characteristic for metastatic cancer consisted of proteins primarily involved in cytokine/chemokine response and/or proliferation.

## Discussion

The last decade has abounded in the publications that reported the MALDI/SELDI-based profiling of the serum peptidome as a promising tool for the effective identification of gastric cancer patients [14–22]. Four different diagnostic classifiers were built on the basis of tri-peptide combination by Ebert et al. [14] (m/z 3946, 3503, 15,958), Su et al. [16] (m/z 1468, 3935, 7560), Liu et al. [19] (m/z 5906.4, 6632.9, 8704.3) and Fan et al. [22] (m/z 1867, 2701, 2094). Although the discriminatory peaks were not consistent among those studies, probably because of the diverse methodology of sample preparation (especially highly abundant proteins removal), measurement and/or data processing [13], there were some common features of proposed signatures. For example, the m/z peak at 1466–1468 Da identified as fibrinopeptide A (FIBA) was reported as a candidate biomarker in three reports [16, 23, 24]. Moreover, increased level of another fragment of FIBA (approx. weight 5904–5906 Da) was observed in serum of patients with gastric cancer [19], but also in serum of patients with ovarian, hepatocellular and urothelial cancers [27–29]. In our study we have detected over 100 components of serum peptidome that differentiate compared groups of gastric cancer patients and healthy volunteers. This included several components that putatively corresponded to fragments of FIBA, exemplified by 1469 and 5904 Da components upregulated in blood of patients with metastatic cancer. Thus, our results clearly confirmed and extended previous reports indicating that multipeptide signatures based on features of endogenous serum peptidome could be used for classification of patients with gastric cancer and differentiation of patients with metastatic disease.

In the second part of our study serum samples were further analyzed using the shotgun LC–MS/MS approach, which is currently the gold standard for identification of proteins allowing label-free quantitation and providing large coverage of sample’s proteome [30]. Among serum proteins with abundances significantly different between healthy individuals and patients with gastric cancer dominated those associated with immunity, inflammation and acute phase response, even though immunoglobulins were excluded from the analysis. Up-regulation of proteins involved in immunity and inflammation is a typical picture of serum proteome of cancer patients, especially in advanced cases, and increased levels of proteins like C-reactive protein, haptoglobin or serum amyloid have been previously reported for many different types of cancer [31–35]. Most recently, iTRAQ-based approach has been used to identify serum proteins differentiating healthy controls and patients with gastric adenocarcinoma in a small sample of Asian population [36]. Upregulation of 48 proteins in samples of cancer patients was reported, that included several inflammation/acute phase-related proteins: A1AG1 (ORM1), A1AT (SERPINA1), AACT (SERPINA3), CO4B, CO9, HPT, ITIH4, LBP and SAA1, which upregulation was revealed also in our study. Moreover, other proteins revealed in both reports included upregulated A2GL (LRG1), ITIH3, ORM2 and SHGB, which collectively indicated very high conformity of serum proteome signature of gastric cancer that based on samples of unrelated Polish and India’s populations.

Moreover, our study revealed serum proteome components with levels discriminating patients with locally advanced gastric adenocarcinoma and with metastatic disease. Proteins upregulated in serum of patients with metastatic disease included C-reactive protein and IC1 (SERPING1), factors involved in immunity and inflammation, and angiogenin, protein involved in angiogenesis. Other proteins differentially expressed in blood of patients with local and metastatic cancer included molecules associated with response to cytokines/chemokines, leukocyte migration and blood coagulation as well as factors associated with extracellular transport, and metabolism of phosphorus and nucleic acids. Elevated level of CEA and CA 19-9 was previously associated with increased risk of gastric cancer metastases. However, increased level of such “classical” markers has been observed only in case of 30–50 % of patients with disseminated disease [3–5], thus there is an obvious space for new proteomics-based markers of metastatic gastric cancer. Furthermore, several patients enrolled into the study were diagnosed with locally advanced cancer, yet in some cases the disease was spread and distant metastases were detected during follow-up. However, comparison of pre-treatment serum samples collected in both subgroups of patients with local disease (who further either developed or did not distant metastases) revealed very few significant differences (similar results were delivered by LC–MS/MS-based analysis of complete proteome and MALDI-based profiling of endogenous peptidome). Hence, our data revealed serum proteome signature discriminating patients with locally advanced and metastatic gastric adenocarcinoma. However, our study did not reveal serum proteome components that could be used for prediction of risk of metastasis in patients diagnosed with local cancer.

## Conclusions

Significant differences between patients with gastric cancer and healthy individuals as well as between patients with locally advanced and metastatic cancer have been detected at the level of serum proteome in pre-treatment blood samples. However, no evidence that features of pre-treatment serum proteome could predict a risk of cancer dissemination in patients treated due to local disease has been observed. Nevertheless, presented data confirmed potential applicability of biomarkers based on serum proteome signature in diagnostics of gastric cancer.

## Additional file

**Additional file 1.** Detailed description of differentiating components of serum proteome.
